# Bovine In Vitro Fertilization Embryo Development Predicted by Capacitation-Induced Zinc Ion Flux and Acrosome Remodeling

**DOI:** 10.3390/ijms27156731

**Published:** 2026-07-28

**Authors:** Kourtney Jimmerson, Marianna Jahnke, Emma Keller, Austin Putz, Daniela Demetrio, Tyler Dohlman, Karl Kerns

**Affiliations:** 1 Department of Animal Science, Iowa State University, Ames, IA 50011, USA; kgrimm20@gmail.com (K.J.); ekel@iastate.edu (E.K.); aputz@iastate.edu (A.P.); 2 Department of Veterinary Diagnostic and Production Animal Medicine, College of Veterinary Medicine, Iowa State University, Ames, IA 50011, USA; marianna@iastate.edu (M.J.); dohlmandvm@gmail.com (T.D.); 3 RuAnn Genetics, Riverdale, CA 93656, USA; ddembryos@gmail.com

**Keywords:** IVF, sperm, capacitation, bovine, zinc signature

## Abstract

Sire-dependent variation in bovine in vitro fertilization (IVF) success is poorly explained by conventional semen parameters once samples meet standard quality thresholds. Sperm capacitation is required for acquisition of fertilization competence, yet its contribution to sire-dependent variation in bovine IVF outcomes remains unclear. Fifteen bulls (*Bos taurus*) were evaluated across five IVF replicates using 30 abattoir-derived oocytes per bull per replicate. Sperm were analyzed before (0 h) and after 3 h of in vitro capacitation using image-based flow cytometry to quantify zinc ion localization patterns (zinc signatures; Fluo-Zin 3 AM), acrosome remodeling (peanut agglutinin conjugated to Alexa Fluor 647; PNA-AF647), and plasma membrane integrity (propidium iodide). Motility parameters were assessed using computer-aided sperm analysis (CASA). Cleavage rate was assessed on Day 3 and blastocyst development on Day 7. Substantial bull-to-bull variation was observed for both cleavage (31.5–76.3%) and blastocyst development (7.3–40%). Bull effects on cleavage were significant (*p* < 0.0001). Predictive modeling revealed that CASA or biomarker variables alone explained limited variance in embryo development. In contrast, models incorporating capacitation-induced changes in zinc signatures and acrosome remodeling significantly improved prediction of IVF outcomes. Integration of biomarker and motility parameters across time points yielded the highest explanatory power, with zinc signature dynamics among the most consistently informative predictors. These findings demonstrate that sperm capacitation-associated zinc flux and acrosomal remodeling are biologically relevant determinants of bull-specific variation in bovine IVF embryo development and provide mechanistic biomarkers that improve prediction beyond conventional motility assessment alone.

## 1. Introduction

Mammalian ejaculates comprise heterogeneous sperm subpopulations that differ in their capacity to undergo capacitation and achieve fertilization competence [1,2,3,4]. Mammalian spermatozoa must undergo capacitation within the female reproductive tract before acquiring the ability to fertilize [5,6]. Single-cell analyses have since revealed that hallmark capacitation events, including membrane potential changes and Ca^2+^ responses, occur in only a subset of sperm under capacitating conditions [2,3]. This heterogeneity is particularly relevant in bull sperm, where cryopreservation can initiate premature capacitation-like changes and alter the distribution of functional states present at the time of fertilization [7,8,9,10,11]. Consequently, conventional sperm metrics such as motility and morphology, while effective for identifying gross defects, have limited power to explain fertility variation among samples that meet standard thresholds, motivating multiparametric assessments of sperm function and capacitation competence [12,13,14,15,16,17].

Capacitation is a temporally staged process driven by coordinated ion fluxes, phosphorylation cascades, and membrane remodeling that culminate in competence for zona binding and acrosomal exocytosis [1,4]. Among candidate signaling ions, Zn^2+^ has emerged as a regulator of sperm function and capacitation, with controlled Zn^2+^ availability influencing motility transitions and acrosomal remodeling [18,19]. Mechanistic studies in bovine sperm show that extracellular Zn^2+^ can stimulate capacitation signaling and the acrosome reaction through zinc receptor/G protein-coupled receptor 39 (ZnR/GPR39)-dependent pathways involving epidermal growth factor receptor (EGFR) activation and downstream kinase signaling [20]. Spermatozoa are ejaculated into seminal plasma that contains the highest Zn^2+^ concentration of any bodily fluid [21,22]. As spermatozoa are separated from seminal fluid and ascend the female reproductive tract, the surrounding Zn^2+^ concentration falls steeply, reaching micromolar levels at the site of fertilization [23]. This decline is permissive rather than inductive: it relieves the constraint imposed by seminal Zn^2+^ and thereby allows, but does not by itself drive, the ordered efflux and intracellular redistribution of sperm zinc, which proceeds as spermatozoa encounter capacitating stimuli within the female reproductive tract [22,23]. Because Zn^2+^ stabilizes sperm chromatin and protects flagellar outer dense fiber thiols from premature oxidation [22], and restrains the ion-channel and proteolytic activities required for hyperactivation and zona pellucida penetration [22,23], its ordered removal is a prerequisite for, rather than a by-product of, capacitation [22,23]. Using image-based flow cytometry, distinct, conserved patterns of sperm Zn^2+^ localization, termed zinc signatures, were shown to shift during in vitro capacitation and respond to perturbations of zinc homeostasis, linking Zn^2+^ redistribution to capacitation-associated remodeling events [21,22,23]. These signatures range from fully zinc-loaded, non-capacitated sperm (signature 1) through progressive zinc efflux states reflecting hyperactivation, binding of oviductal glycans, acrosome remodeling, zona pellucida binding, and acrosome exocytosis (signatures 2–4), with bull spermatozoa displaying an additional acrosome-localized fifth pattern [22]. Consistent with this progression, only spermatozoa bearing signatures 1 and 2 bound glycans of the oviductal sperm reservoir [23], and among these, hyperactivated spermatozoa capable of recognizing and binding the zona pellucida were observed to carry signature 2 [21]. Signatures 3 and 4 did not bind these glycans, instead representing the late and complete stages of capacitation, with acrosomal membrane remodeling in both and spontaneous acrosomal exocytosis occurring predominantly in signature 4 [21,23]. Proteomic studies further support Zn^2+^ as a broad regulator of sperm function during capacitation [24].

In vitro embryo production now accounts for approximately 80% of all transferable bovine embryos worldwide [25], yet bull-to-bull variation in cleavage and blastocyst development remains difficult to predict from routine semen evaluation. Prior work indicates that functional panels integrating membrane and acrosome integrity, mitochondrial function, and motility traits can associate with bovine IVF embryo development outcomes [26,27], and multiparametric flow cytometry approaches improve fertility prognostics relative to single measures [15,28]. Although zinc signature dynamics have been linked to artificial insemination (AI) field fertility differences among bulls [29] and boars [21], AI pregnancy rate and IVF embryo development rate reflect fundamentally different biological endpoints in that IVF requires sperm to capacitate without the selective environment of the female reproductive tract. Direct tests connecting capacitation-induced zinc redistribution to bovine IVF embryo development have not been conducted. Recently, we demonstrated that capacitation-induced changes to sperm zinc signatures also predict porcine IVF outcomes [30], underscoring the cross-species relevance of capacitation-associated zinc dynamics as fertility biomarkers. Importantly, delta changes in biomarker profiles between pre- and post-capacitation timepoints may capture the functional responsiveness of the sperm population to capacitation stimuli, a dynamic dimension absent from conventional single-timepoint assessments [15,29]. We therefore hypothesized that capacitation-induced changes in zinc signatures, together with acrosomal remodeling and membrane integrity, would predict bull-specific differences in IVF outcomes beyond conventional semen quality measures; specifically, that sperm samples retaining a pre-capacitation profile at baseline would outperform those exhibiting premature capacitation, and that the magnitude of capacitation-induced changes would be a stronger predictor than any single time-point measurement.

## 2. Results

### 2.1. Embryo Development Endpoints and Bull Variation

**Figure 1:**
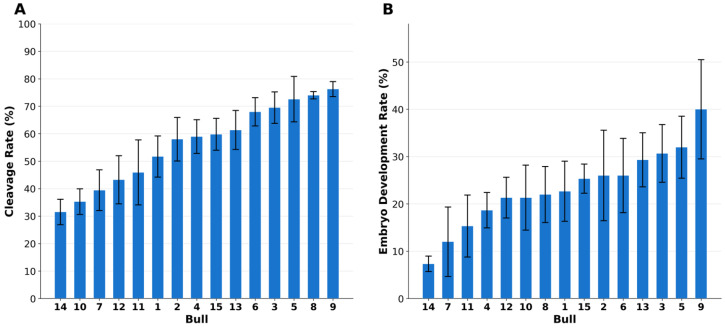
Bull-specific cleavage and blastocyst development rates across replicates. (**A**) Mean cleavage rate (CR, Day 3) for each bull averaged across five IVF replicates. (**B**) Mean embryo development rate (EDR, Day 7 blastocyst rate) for each bull averaged across five IVF replicates. Error bars represent ± standard error of the mean (SEM) (*n* = 5 replicates per bull). For each replicate, 30 MII oocytes were fertilized per bull, for a maximum of 150 oocytes evaluated per bull across the study. Bulls are ordered from lowest to highest mean value within each endpoint. Descriptive statistics for each bull are provided in Supplemental Table S1. Bull significantly affected cleavage rate (*p* < 0.0001) but not blastocyst rate (*p* = 0.08; two-way ANOVA with IVF replicate as a block).

Five embryo development endpoints were evaluated: maturation (MAT) to Day 7, in vitro culture (IVC) to Day 7, cleavage to Day 7, MAT to cleavage, and IVC to cleavage. Where IVC denotes the time point immediately after cumulus stripping, cleavage was assessed on Day 3, and embryo development on Day 7. The Day 7 developmental endpoint was defined as the proportion of embryos reaching the blastocyst or expanded blastocyst stage on Day 7 of culture. Specifically, MAT to Day 7 expresses Day 7 blastocysts as a proportion of matured (metaphase II, MII) oocytes; IVC to Day 7, as a proportion of presumptive zygotes placed into culture after cumulus stripping; and cleavage to Day 7, as a proportion of cleaved embryos on Day 7. MAT to cleavage and IVC to cleavage express Day 3 cleavage relative to matured and cultured oocytes, respectively. Descriptive statistics for each response are summarized in Table 1. Substantial bull-to-bull variation was observed across replicates (Figure 1A,B), with cleavage ranging from 31.5% to 76.3% and Day 7 blastocyst development from 7.3% to 40% (Supplemental Table S1). The bull significantly affected the cleavage rate (*p* < 0.0001) but not the blastocyst rate (*p* = 0.08; two-way analysis of variance (ANOVA) with IVF replicate as a block; Figure 1).

### 2.2. Zinc Signature Classification by Image-Based Flow Cytometry

**Figure 2:**
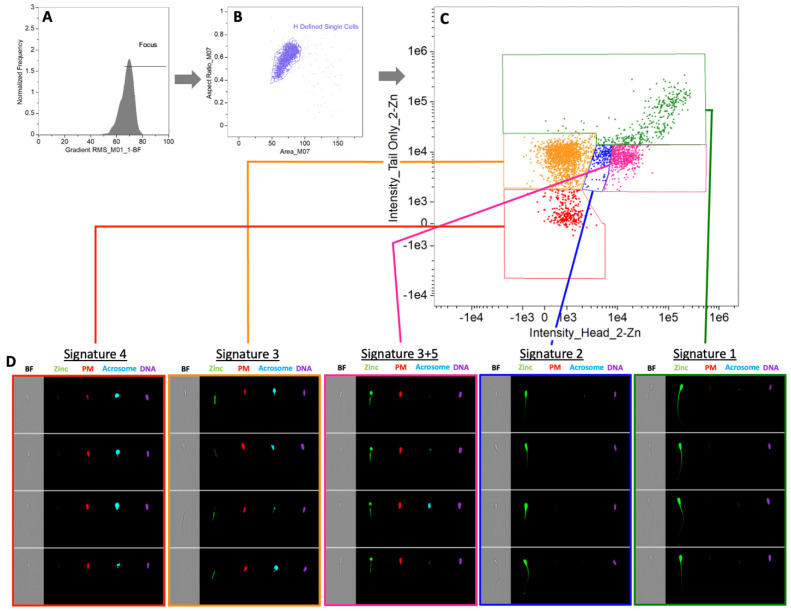
Gating strategy and zinc signature classification by image-based flow cytometry. (**A**) Representative histogram showing focus gating of brightfield images using Gradient RMS to exclude out-of-focus events. (**B**) Single-cell discrimination based on area and aspect ratio of Hoechst 33342 (Area_M07 × Aspect Ratio_M07) to select single spermatozoa per image. (**C**) Bivariate plot of zinc probe intensity in the sperm head (Intensity_Head_2-Zn) versus tail (Intensity_Tail_Only_2-Zn) used to define zinc signature populations (Signatures 1, 2, 3, 3 + 5, and 4). Gates are colored by signature: Signature 1, green; Signature 2, blue; Signature 3 + 5, pink; Signature 3, orange; Signature 4, red. The same colors outline the corresponding image galleries in (**D**). (**D**) Representative image galleries for each zinc signature population. Images include brightfield (BF), zinc indicator FluoZin-3 AM (Zinc, green), propidium iodide for plasma membrane integrity (PM, red), PNA-AF647 for acrosome status (Acrosome, cyan), and Hoechst 33342 for nuclear DNA/sperm head (DNA, blue). The rationale and criteria used to define the zinc signature populations are described in the Materials and Methods (Section 4.9).

Sperm zinc localization patterns were assessed at 0 h and 3 h of in vitro capacitation using image-based flow cytometry. Focused, single spermatozoa were identified by gradient root mean square (RMS) and morphometric gating (Figure 2A,B), and zinc signature subpopulations were resolved on a bivariate plot of head versus tail zinc probe intensity (Figure 2C). Subpopulation gating by PNA-AF647 (Invitrogen^TM^; Eugene, OR, USA) intensity, reflecting acrosome remodeling status, was required to resolve discrete zinc signatures. All five zinc signatures were present at both time points, with capacitation inducing redistribution across signature classes as visualized by Fluo-Zin 3 AM (Thermo Fisher; Waltham, MA, USA) intensity plots and corresponding image galleries (Figure 2C,D).

### 2.3. Correlation Structure Among Biomarkers and Motility Parameters

**Figure 3:**
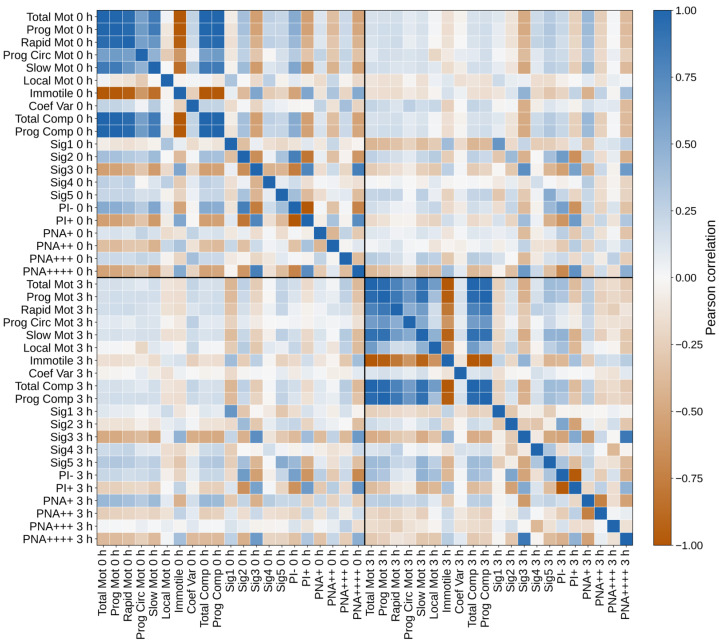
Correlation heatmap of biomarkers and CASA motility parameters across time points. Pairwise Pearson correlations were calculated among zinc signature subpopulations, plasma membrane integrity (PI), acrosome status (PNA), and CASA kinematic variables measured at 0 h and 3 h of in vitro capacitation. Cells show correlation coefficients (scale: 1.0, strong positive, to −1.0, strong negative), with darker blue indicating positive associations and darker orange indicating negative associations. Variables are grouped by assay and time point. Abbreviations: Total Mot, total motility; Prog Mot, progressive motility; Rapid Mot, rapid motility; Prog Circ Mot, progressive circular motility; Slow Mot, slow motility; Local Mot, local motility; Immotile, immotile sperm; Coef Var, coefficient of variation; Total Comp, total composite score; Prog Comp, progressive composite score. Sig1–Sig5 denote zinc signatures 1–5; PI− and PI+ denote propidium iodide-negative and positive spermatozoa (plasma membrane integrity); PNA+ through PNA++++ denote increasing peanut agglutinin (acrosome) labeling; 0 h and 3 h indicate the pre- and post-capacitation timepoints.

Pairwise correlations among zinc signatures, plasma membrane integrity (PI), and acrosome status (PNA), as well as CASA kinematic parameters at 0 h and 3 h, revealed distinct clustering by assay type (Figure 3). CASA velocity and motility parameters clustered tightly within each time point, and biomarker variables correlated with one another, particularly among zinc signature subclasses and acrosome states. Correlations between biomarker and motility profiles were weaker and more variable, with both positive and negative associations. Notably, correlations between 0 h and 3 h measures were often weak or reversed in direction, indicating that pre-capacitation values did not reliably predict post-capacitation profiles and underscoring the information gained from measuring at both timepoints.

### 2.4. Predictive Modeling of Embryo Development

**Figure 4:**
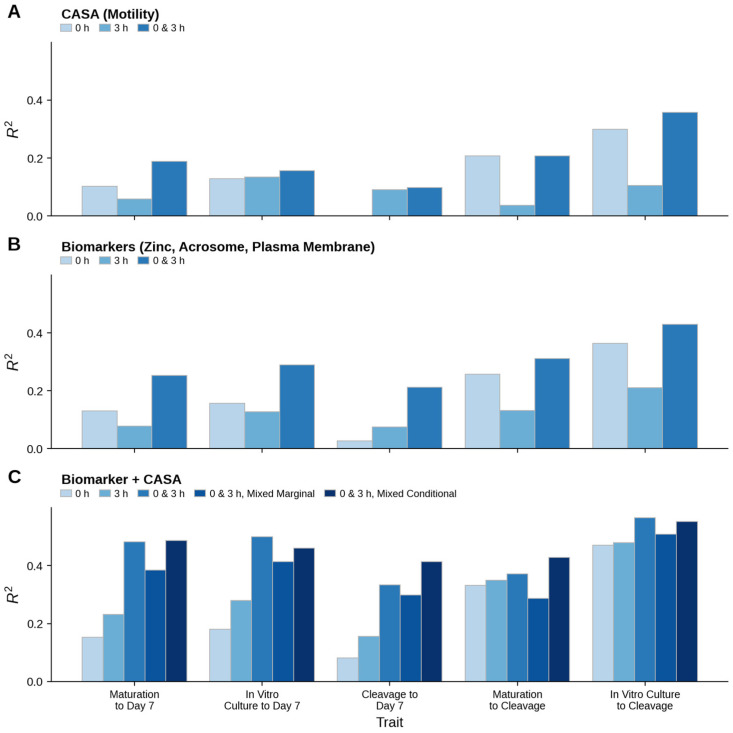
Comparison of predictive models for five embryo development endpoints, shown as the coefficient of determination (R^2^) from OLS regression with stepwise Akaike information criterion (AIC)-selected predictors. Panels group models by parameter: (**A**) CASA motility parameters; (**B**) biomarkers (zinc signatures, acrosome remodeling [PNA], and plasma membrane integrity [PI]); and (**C**) both combined. Within each panel, bars show models built from baseline (0 h) parameters, post-capacitation (3 h) parameters, and both timepoints (0 & 3 h); for the combined model in (**C**), marginal and conditional R^2^ from linear mixed models (bull and replicate as random effects) are also shown. The five endpoints are maturation to Day 7, IVC to Day 7, cleavage to Day 7, maturation to cleavage, and IVC to cleavage. Statistics are provided in Supplemental Table S2.

Predictive modeling revealed a consistent hierarchy of explanatory power across endpoints (Figure 4; Supplemental Table S2). CASA or biomarker variables alone explained limited variance in embryo development. Incorporation of 3 h post-capacitation data improved model fit, and integration of both time points yielded the highest R^2^ values (R^2^ = 0.33–0.56). For maturation to Day 7, the endpoint in which the denominator of matured oocytes most directly isolates the sperm contribution to blastocyst development, combined parameters at 0 h alone yielded an R^2^ of 0.153, rising to 0.482 when capacitation-induced changes were included. R^2^ values were highest for IVC-to-cleavage and lowest for cleavage-to-Day 7 across all model types. Mixed models produced marginal and conditional R^2^ values comparable to or exceeding ordinary least squares (OLS) estimates, with conditional R^2^ being consistently highest, confirming that both measured functional traits and residual bull-level variance contributed to developmental outcomes. When each assay was examined alone at a single timepoint (Figure 4A–C), no single domain at a single timepoint explained substantial variance; CASA at 0 h was the weakest predictor (R^2^ = 0.00–0.30), biomarkers exceeded CASA at each timepoint, and the largest gains arose when both timepoints, and especially both domains, were combined. Formal nested (partial F) tests confirmed that adding the capacitation-induced change (Δ, 0 → 3 h) predictors significantly improved model fit for all five endpoints (maturation to Day 7, F(9, 55) = 3.52, *p* = 0.002; IVC to Day 7, F(10, 56) = 3.35, *p* = 0.002; cleavage to Day 7, F(9, 57) = 3.71, *p* = 0.001; maturation to cleavage, F(4, 65) = 2.65, *p* = 0.041; IVC to cleavage, F(5, 61) = 3.36, *p* = 0.010). Full test statistics are provided in Supplementary Table S4.

### 2.5. Bull Repeatability

**Figure 5:**
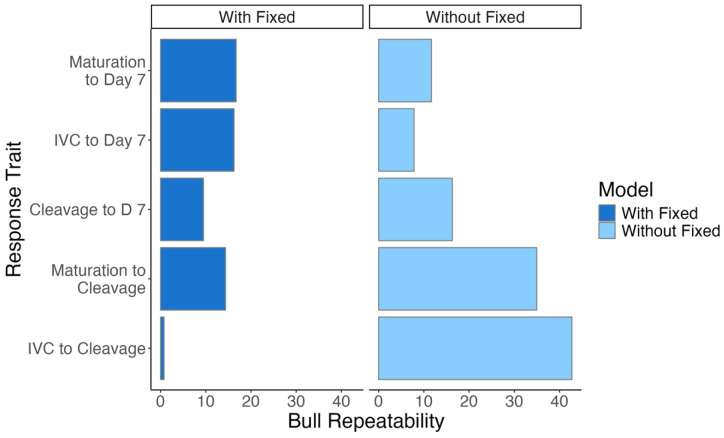
Bull repeatability across embryo development endpoints. Repeatability of the bull effect (calculated as described in Materials and Methods) for each response variable across five IVF replicates. Dark blue bars represent repeatability from mixed models including stepwise AIC-selected CASA and biomarker covariates; light blue bars represent repeatability from intercept-only models with bull as the sole random effect. A reduction in repeatability after including the stepwise-selected CASA and biomarker covariates indicates that these variables account for part of the between-bull variability in the corresponding endpoint, whereas repeatability that remains high indicates additional bull-specific factors not captured by the current biomarker panel.

Bull repeatability varied across endpoints (Figure 5). Before accounting for sperm parameters, repeatability was highest for IVC-to-cleavage and maturation-to-cleavage, reflecting substantial sire-dependent variance. Inclusion of CASA and biomarker predictors markedly reduced repeatability, most notably for IVC-to-cleavage, demonstrating that zinc signatures and acrosome remodeling metrics captured a meaningful proportion of previously unexplained bull variance. Endpoints for which repeatability remained elevated after covariate adjustment suggest the contribution of additional sire-level factors beyond the current biomarker panel. Biologically, repeatability represents the fraction of variation attributable to differences among bulls after accounting for the modeled fixed effects; a decrease in repeatability upon adding CASA and biomarker covariates indicates that these variables explained part of the between-bull variation, whereas repeatability that remained elevated indicates additional sire-specific factors not captured by the current panel.

### 2.6. Variable Importance

**Figure 6:**
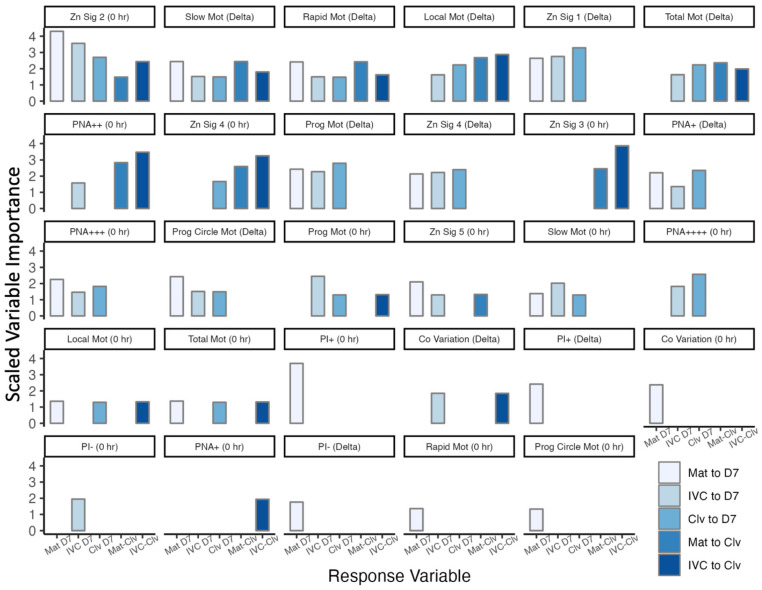
Variable importance of predictors across embryo development endpoints. Variable importance for each predictor retained by stepwise AIC selection in the combined CASA-plus-biomarker OLS models. Each panel represents one predictor, with up to five bars corresponding to the five response variables. Predictors are ordered by frequency of retention across models. Numeric values are provided in Supplemental Table S3. Motility, zinc signature (Sig), plasma membrane integrity (PI), and acrosome (PNA) abbreviations follow those defined for Figure 3; 0 h denotes the baseline (thaw) value and ‘Delta’ the capacitation-induced change (0 → 3 h).

Variable importance analysis identified capacitation-responsive traits as the most consistent contributors to model performance (Figure 6; Supplemental Table S3). The capacitation-induced change in zinc signature 1 (Signature 1 Delta), reflecting the proportion of sperm exiting the non-capacitated state, and the baseline prevalence of signature 2 at 0 h, an intermediate capacitation state, ranked among the top predictors across multiple endpoints. Acrosome remodeling indicators (PNA++ at 0 h, PNA+ Delta) and capacitation-associated motility shifts, particularly in progressive and local motility, also contributed substantially, with relative importance varying by developmental stage. Static 0 h motility measures contributed comparatively less and in a more response-specific manner.

## 3. Discussion

This study demonstrates that integrating sperm zinc signatures and acrosome remodeling biomarkers with conventional CASA motility parameters substantially improves the prediction of bull-specific in vitro fertilization embryo development outcomes compared to either approach alone. This finding is consistent with prior work demonstrating that single semen parameters have limited predictive value once basic quality thresholds are met [12,14,15]. For maturation to Day 7, the endpoint most reflective of the sperm’s contribution to blastocyst development, neither CASA parameters at 0 and 3 h (R^2^ = 0.188) nor biomarker parameters at 0 and 3 h (R^2^ = 0.253) alone adequately predicted embryo development, and combining both domains at a single timepoint offered limited improvement (R^2^ = 0.153 at 0 h; R^2^ = 0.231 at 3 h). Only when motility and biomarker data were integrated across both timepoints, capturing capacitation-induced changes, did explanatory power substantially increase (R^2^ = 0.482). Across all five endpoints, combined models achieved R^2^ values of 0.33–0.56.

The consistent finding across all endpoints was that no single domain or single timepoint was sufficient (Figure 4; Supplemental Table S2). CASA captures sperm kinematics but does not assess acrosome integrity or capacitation status; biomarker data captures functional state, but not motility; and parameters measured at only one timepoint miss the dynamic changes that distinguish fertile from subfertile bulls. The requirement for all four components (motility, biomarkers before and after intentional capacitation) underscores that sire fertility is a multivariate, time-dependent trait, as suggested by previous multiparametric fertility models in cattle [15,28]. Variable importance analysis revealed that capacitation-responsive biomarkers, particularly zinc signatures and acrosome remodeling indicators (PNA++ at 0 h and PNA+ Delta), were consistently among the top predictors across multiple response variables. Motility parameters, particularly changes in progressive and total motility during capacitation, also ranked highly, consistent with the known importance of hyperactivated motility for zona pellucida penetration. When each domain was modeled alone (Figure 4A,B), biomarkers outperformed CASA at every endpoint, and CASA at 0 h was the weakest single predictor (R^2^ = 0.00–0.30); the largest gains emerged only when both timepoints, and then both domains, were combined (Figure 4C), reinforcing that capacitation dynamics, rather than any single static measure, drive predictive power.

The coefficient directions observed in our predictive models are consistent with a capacitation-competence framework in which the functional capacity of sperm to undergo ordered capacitation events, rather than their state at any single time point, determines fertilization success. Following the capacitation progression described for zinc signatures [21], the baseline zinc profile was informative: signature 2 at 0 h, representing an early intermediate capacitation state, was the single strongest biomarker predictor across multiple endpoints (β = −2.53, *p* < 0.001 for blastocyst development). Its biological interpretation is complicated by the use of cryopreserved semen; in fresh semen, signature 2 represents a pre-capacitation zinc state, but cryopreservation-induced capacitation-like changes may shift its meaning, as cryo-induced membrane destabilization and premature capacitation have been well documented in bovine sperm [7,8,9,10]. Signature 2 retention at baseline reflects sperm that are partially cryo-capacitated but have not progressed beyond this physiological point in the sperm capacitation sequence. Conversely, elevated proportions of sperm exhibiting late-stage zinc signatures (signatures 3 and 4) or advanced acrosome remodeling (high PNA++/+++/++++, low PNA+) at 0 h were negatively associated with development, consistent with premature capacitation or cryo-capacitation reducing the window of fertilization competence or capacitated prior to insemination. Given that zinc flux has been mechanistically linked to capacitation signaling and acrosome responsiveness in bovine sperm [20,21], excessive baseline redistribution likely reflects functional advancement beyond the optimal fertilization window. At baseline, sperm samples with intact plasma membranes (low PI+) and intact acrosomes (high PNA+, low PNA++/+++/++++) were associated with superior IVF outcomes. The positive coefficient for PI+ change during incubation (β = +2.96, *p* = 0.019), after controlling for baseline PI+, is notable: while PI+ is conventionally interpreted as a marker of membrane damage, capacitation involves substantial plasma membrane reorganization, including cholesterol efflux and increased membrane fluidity, which may transiently increase PI permeability without reflecting true cell death [1,4].

The importance of capacitation-induced changes (Delta values) further supports this interpretation. Following the zinc signature progression, Signature 1 Delta was consistently a negative predictor, suggesting that rapid loss of the non-capacitated state during the 3 h incubation reflects disordered rather than productive capacitation. Similarly, Signature 4 Delta was negatively associated with blastocyst development, indicating that rapid progression to the most capacitated state is also detrimental. In contrast, a positive association between Signature 3 Delta and cleavage rate indicates that controlled progression toward an intermediate-to-capacitated zinc profile is beneficial, consistent with a Goldilocks zone in which sperm must progress through capacitation but not exhaust their fertilization competence prematurely. A similar association between controlled zinc signature transitions and fertility has been reported in AI field fertility differences among bulls [29] and in porcine IVF systems [30], supporting the broader relevance of zinc-responsive capacitation dynamics. Motility parameters reinforced this capacitation-competence model. Progressive motility at baseline was a positive predictor, consistent with healthy forward-swimming sperm entering the fertilization system. However, the change in progressive motility during incubation was negatively associated with outcomes, while increases in slow and local motility were positive predictors. This pattern is characteristic of the hyperactivated motility transition that accompanies capacitation, in which sperm shift from linear, progressive swimming to high-amplitude, asymmetric flagellar beating, a hallmark of fertilization competence described across mammalian species [1,4]. Bulls whose sperm underwent this transition most robustly produced more embryos.

Bull repeatability varied across endpoints, with the largest reduction observed for IVC-to-cleavage upon inclusion of CASA and biomarker covariates (Figure 5), indicating that the biomarker panel captured a substantial proportion of sire-dependent variance for this endpoint. That repeatability remained elevated for other endpoints after covariate adjustment suggests that additional sire-level factors contribute to developmental outcomes beyond what the current panel measures.

Zinc ions are increasingly recognized as molecular regulators of sperm capacitation and fertilization competence, providing a mechanistic basis for the associations reported here. Mammalian spermatozoa acquire a large, predominantly prostate-derived zinc pool, and the controlled efflux and intracellular redistribution of this zinc during capacitation is required for the ordered progression toward hyperactivation, oviductal and zona-pellucida binding, and acrosomal exocytosis [19,21,22,23,24]. Using image-based flow cytometry, conserved patterns of sperm zinc localization (zinc signatures) have been resolved that shift progressively during in vitro capacitation, from a fully zinc-loaded, non-capacitated state (signature 1) through intermediate efflux states (signatures 2 and 3) to the most capacitated state (signature 4) [21,23]. In boar spermatozoa, this progression follows signatures 1 → 2 → 3 → 4, whereas bull spermatozoa additionally display a midpiece and acrosome-localized zinc population, resolved here as the signature 3+5 population, that was present in earlier bovine data although not formally designated [23]. Mechanistically, mobilized Zn^2+^ can stimulate capacitation signaling and acrosome exocytosis in bull sperm through a zinc-sensing G-protein-coupled receptor (ZnR/GPR39) and downstream EGFR and kinase activation [20], and zinc has been described as a master regulator of sperm function during capacitation [24]. The acrosome-localized zinc signature observed in bull spermatozoa, together with its association with embryo development, points to a functional role for a zinc-binding acrosomal receptor/protein in coordinating capacitation-associated acrosome remodeling in bull sperm. Within this framework, the capacitation-induced changes in zinc signatures measured here likely report the capacity of a sperm population to execute this ordered zinc redistribution, consistent with our observation that the magnitude of change (Δ), rather than any single static measurement, was the most informative predictor of embryo development.

Several considerations temper the interpretation of these models. The best combined models explained approximately one-third to one-half of the variance in embryo development (R^2^ = 0.33–0.56), indicating that the biomarker and motility panel captures a meaningful but partial component of sire-dependent variation. Bovine IVF outcomes are multifactorial and are influenced by additional factors not measured here, including oocyte quality and donor variation, sperm DNA and chromatin integrity, centriolar and paternal-genomic contributions, seminal-plasma and epididymal factors, and culture conditions, as well as residual technical and biological variation across replicates not captured by the measured biomarkers. Accordingly, the present models are best interpreted as tools for ranking and screening sires rather than as deterministic predictors of individual embryo outcomes. Validation in larger, genetically diverse bull cohorts, and integration of these capacitation-responsive biomarkers with field fertility and complementary sperm-quality measures, will be needed to establish their value for routine sire selection.

## 4. Materials and Methods

### 4.1. Study Design

This study used fifteen bulls (*Bos taurus*), selected to represent a range of expected fertility, for each replicate across five replicates. For each replicate, each bull had the capacity to fertilize thirty MII abattoir oocytes. Thus, over the course of the five replicates, each bull had the capacity to fertilize up to 150 oocytes.

### 4.2. Media Used for IVF

Media used in this study for in vitro production (IVP) embryos was produced by a commercial laboratory (RuAnn Genetics, Riverdale, CA, USA). Ovoil™ (Vitrolife Sweden AB, Västra Frölunda, Sweden) was used to prevent evaporation of the IVF media while in the incubator. Maturation was carried out in supplemented tissue culture medium 199 (TCM-199) and fertilization in supplemented Tyrode’s albumin lactate pyruvate (TALP) medium, and embryo culture used supplemented synthetic oviduct fluid (SOF) medium for both the IVC-D3 and IVC-D5 stages, as described previously [31]. Heparin was included in the fertilization medium to support sperm capacitation. All maturation, fertilization, and in vitro culture (IVC-D3 and IVC-D5) media were obtained ready-to-use from the commercial provider (RuAnn Genetics) and were used as supplied; no additional media components were prepared or supplemented in our laboratory. The formulations indicated above reflect the commercial media used.

### 4.3. Processing of Oocytes

Oocytes were obtained from abattoir ovaries from dairy cows (*Bos taurus*), and the source was consistent across all bulls and IVF replicates. The oocytes were shipped in maturation media in an incubator at 38.5 °C overnight to Iowa State University. Upon arrival, the incubator temperature was checked and oocytes were removed. The oocytes were then placed in new maturation media in a 4-well NUNC dish and placed in an incubator (38.7 °C, 5% CO_2_ in a humidified atmosphere) with a maximum of 35 cumulus-oocyte complexes (COCs) per drop. The oocytes were in maturation media for 18–24 h before being washed once with fertilization media and then placed in fertilization media with 30 oocytes per well. Once placed in fertilization media, the oocytes were placed back in the CO_2_ incubator until the sperm were processed and ready for fertilization.

### 4.4. Semen Processing

Semen for fertilization was thawed in a water bath set at 37 °C and then placed into a 1.5 mL microcentrifuge tube. A motility assessment was done before the sperm cells were centrifuged. Approximately half of the semen was then placed carefully on top of two layers (40 and 80%) of silica-based colloidal medium (Puresperm, Nidacon International AB, Gothenburg, Sweden). The other half of the semen was used for the 0 h control sample. Semen was then centrifuged for five minutes at 2160× *g* force to separate the live spermatozoa from the cryoprotectant, seminal plasma, and dead spermatozoa. After the first centrifugation, the supernatant was removed from the pellet and 500 µL of equilibrated in vitro fertilization media was placed with the pellet. Sperm were then centrifuged again for three minutes at 780× *g* force. After the second centrifugation, the supernatant was removed again from the pellet, and 100 µL in vitro fertilization media was added. The sperm concentration was then taken using a NucleoCounter SP-100 (ChemoMetec, A/S, Allerød, Denmark), and calculations were determined for insemination using a previous protocol [31]. A single straw was thawed per bull for each replicate, and a single ejaculate per bull was used throughout the study. The oocytes were inseminated with 10–25 µL of the prepared sperm suspension to achieve a final concentration of approximately 2 × 10^6^ sperm/mL. All motility and biomarker analyses were performed on washed, Puresperm-selected sperm, consistent with the fraction used for fertilization. A single cryopreserved ejaculate was used for each bull throughout the study, with one straw thawed per replicate; thus, the conditions were held constant within each bull. Post-thaw sperm parameters (including total and progressive motility and acrosome [PNA] status) were measured in every replicate and showed replicate-to-replicate variation within bulls; this replicate-level variation was accounted for by including replicate as a random effect in the mixed models.

### 4.5. In Vitro Fertilization

After sperm were prepared for fertilization, the oocytes were fertilized and placed back in the CO_2_ incubator. After 8–12 h of coincubation with sperm, the oocytes were analyzed, and cumulus cells were stripped by pipetting. The oocytes were rinsed with culture media and then placed into 70 µL culture media drops overlaid with 1000 µL oil in 4-well NUNC dishes and then placed in a tri-gas incubator (38.5 °C, 6% CO_2_, 5% O_2_ in a humidified atmosphere). Zygotes were analyzed 70–72 h (D3) after insemination to analyze for cleavage rates and 65 µL culture media was replaced with 65 µL fresh IVC-D3 media. Any zygotes not cleaved were left in the culture media to get an accurate count at the end of the seven days. On day 5, 65 µL IVC-D3 media was removed from each well and replaced with 65 µL fresh IVC-D5 media [31]. Day 7 embryos were analyzed for stage and quality using the International Embryo Technology Society (IETS) standards [31]; embryos reaching the blastocyst or expanded blastocyst stage on Day 7, with a defined inner cell mass and distinct trophoblast cells, were scored as developed [31]. The remaining prepared sperm from each bull was incubated in fertilization medium for 3 h and then analyzed; thus, the 3 h capacitation time point reflects this residual sperm aliquot rather than a pre-incubation of sperm before insemination.

### 4.6. Reagents for Zinc Signature Analysis

Primary stocks of fluorescent probes were produced. These stocks included Lectin PNA (Arachis hypogaea/peanut agglutinin) conjugated to Alexa Fluor™ 647 from Invitrogen (L32460; Eugene, OR, USA) at a stock concentration of 1 mg/mL. Fluo-Zin™3, AM (FZ3; zinc probe) from Thermo Fisher (F24195; Waltham, MA, USA) was reconstituted with dimethyl sulfoxide (DMSO) to a stock solution for 500 µg/mL. Hoechst 33342 (H33342) from Invitrogen (H1399; Eugene, OR, USA) was reconstituted with ddH_2_O to a stock solution of 18 mM. Propidium Iodide from Invitrogen (P304MP; Eugene, OR, USA) was reconstituted with H_2_O to a stock solution of 1 mg/mL.

### 4.7. Zinc Signature Staining

Non-capacitating samples (0 h) were prepared for image-based flow cytometry (IBFC) acquisition while the in vitro capacitation samples were incubated for 3 h in IVF media and a tri-gas incubator. A total of 100 µL (5 million sperm cells) was taken from each sample and rinsed with non-capacitating media (NCM), supernatant was removed, and spermatozoa were resuspended with 100 µL of secondary probe containing: FZ3 (1:500 dilution of stock; final concentration of 1 µg/mL), PNA-AF647 (1:2000; 0.5 µg/mL), PI (1:1000; 0.1 µg/mL), and H33342 (1:1000; 10 µg/mL). Samples were incubated at 37 °C for 30 min with a probe covered with aluminum foil to protect it from the light. After 30 min, samples were spun at 500 g force for 4 min, the supernatant was removed, and spermatozoa were resuspended with 100 µL phosphate-buffered saline (PBS) with no sodium azide. PBS was added, and samples were incubated for another 30 min at 37 °C and covered with aluminum foil to allow the de-esterification of FZ3 as per manufacturer protocol. Next, samples were imaged on the ImageStreamX Mark II image-based flow cytometer immediately following the second 30 min incubation. These steps were repeated for the samples undergoing in vitro capacitation.

### 4.8. Image-Based Flow Cytometry (IBFC) Acquisition

Sperm cells were imaged using the ImageStreamX^®^ Mark II (AMNIS Cytek Biosciences, Fremont, CA, USA) image-based flow cytometer, fitted with 20×, 40× and 60× objective lens magnifications and a 405 nm, 462 nm, 488 nm, 561 nm, and 785 nm laser. Images were acquired using a template with gating to collect 50,000 cells in focus. The images were collected with 40× magnification. Lasers used to acquire images were 405 nm (for H33342; 10.00 mW), 488 nm (for FZ3 and PI; 60.00 mW), 642 nm (for PNA-AF647; 25.00 mW), and 785 nm (for side scatter; 1.00 mW).

### 4.9. IBFC Data Analysis

Data were analyzed using Amnis IDEAS^®^ version 6.3 software (AMNIS Cytek Biosciences, Fremont, CA, USA). The gating approach included a strategy to analyze only cells that were in focus, single cells per image, and those that were anteriorly/posteriorly aligned with the camera (as opposed to laterally aligned) as previously described [21]. Boundaries for gating sperm populations were determined by population segregation and using the image gallery. Zinc signature populations were defined following the classification established for mammalian sperm [21,23], in which capacitation-associated zinc efflux produces conserved, progressive localization patterns. Populations were resolved on a bivariate plot of zinc-probe intensity in the sperm head versus tail (Figure 2C) and imaging from IBFC validated gating boundaries. Zinc signatures were used because the ordered efflux and intracellular redistribution of Zn^2+^ is itself a prerequisite for, and gatekeeper of, capacitation rather than a correlate of it [22], and spermatozoa within an ejaculate progress through it heterogeneously [21,23]; single-cell imaging of zinc localization therefore resolves discrete functional subpopulations that population-average measurements would obscure, using localization patterns conserved in boar, bull, and human spermatozoa [21]. The signatures were assigned as follows: signature 1, fully zinc-loaded (non-capacitated); signature 2, early intermediate efflux; signature 3, advanced efflux; and signature 4, most capacitated. Bull spermatozoa additionally display a midpiece with acrosome-localized zinc population; the “3+5” population resolved here corresponds to this bull-specific acrosomal distribution, which was present in earlier bovine data although not formally termed [23].

### 4.10. Statistical Analysis

Differences among bulls in cleavage and blastocyst development rates (Figure 1) were assessed by two-way analysis of variance (ANOVA) with bull and IVF replicate (block) as factors (α = 0.05). Five response variables were analyzed: maturation to day 7, IVC to day 7, cleavage to day 7, maturation to cleavage, and IVC to cleavage. Both ordinary least squares (OLS) regression and mixed models (with bull and replicate as random effects) were used. All analyses were conducted in R (v4.3.1; R Core Team 2023). OLS models were fit using lm from the stats package, and mixed models were fit using lmer from the lme4 package [32].

Predictors were grouped into biological categories: CASA variables, biomarker variables (zinc signatures, plasma membrane integrity (PI), and acrosome remodeling (PNA)), and a combination of both (described in detail elsewhere in this manuscript). The 0 h and 3 h CASA time points were analyzed separately. For all biomarker and CASA variables, delta values were calculated as the change from 0 h to 3 h (3 h − 0 h), representing capacitation-induced shifts in sperm subpopulation distribution or motility parameters. Pairwise Pearson correlation coefficients were computed among all biomarker and CASA variables at each time point to characterize the inter-variable association structure. Within each category, all predictors were fit and then reduced via stepwise AIC selection (k = 2) from the MASS package [33]. For mixed models, after identifying the best fixed-effect model (variables), a final selection step was run to determine the best model after accounting for bull and replicate as random effects. Model performance was assessed using the performance package [34]. Marginal and conditional R^2^ values [35] were extracted from mixed models, representing variance explained by fixed effects alone and by fixed plus random effects, respectively. Variable importance was calculated using the varImp function from the caret package [36], which standardizes coefficients and scales them from 0 to 100. To formally test whether incorporating capacitation-induced changes improved model fit, each stepwise-selected combined (0 + 3 h) model was compared with a reduced model retaining only its baseline (0 h) terms using a partial (nested) F-test.

Repeatability was calculated to determine the proportion of variance explained by the bull effect. Repeatability here denotes the proportion of total variance attributable to differences among bulls (the bull random effect) after accounting for the fixed effects included in the model; higher repeatability indicates a larger sire-specific contribution to the outcome that is not explained by the modeled covariates.

Repeatability = *σ*^2^_bull_/(*σ*^2^_bull_ + *σ*^2^_rep_ + *σ*^2^_e_)

where *σ*^2^_bull_ is the between-bull variance, *σ*^2^_rep_ the replicate variance, and *σ*^2^_e_ the residual variance.

## 5. Conclusions

In conclusion, combining sperm zinc signature analysis and acrosome remodeling biomarkers with CASA motility parameters across pre- and post-in vitro capacitation timepoints was associated with improved prediction of bovine IVF embryo development rates, extending prior multiparametric fertility models by incorporating dynamic capacitation responsiveness as a predictive dimension [15,29].

## Figures and Tables

**Table 1:** Descriptive statistics for embryo development endpoints. Summary statistics for each of the five embryo development response variables across 15 bulls evaluated over five IVF replicates (30 MII oocytes per bull per replicate; 75 total observations). Values represent percentages.

Min	Q3	Q1	Max	Median	SD	Mean	Response
0	31.7	10	70	20	15.4	23.3	Maturation to Day 7
0	36.9	15	77.8	23.3	17.2	26.6	In vitro culture (IVC) to Day 7
0	60	30	100	43.8	24.1	45.7	Cleavage to Day 7
6.7	63.3	33.3	86.7	53.3	18.2	49.8	Maturation to Cleavage
7.7	72.3	37.5	92.9	60.7	19.7	56.4	In vitro culture (IVC) to Cleavage

Mean, arithmetic mean; SD, standard deviation; Min, minimum; Q1, first quartile; Q3, third quartile; Max, maximum. Values are percentages across 15 bulls and 5 replicates (*n* = 75 observations).

## Data Availability

The original contributions presented in this study are included in the article/Supplementary Material. Further inquiries can be directed to the corresponding author.

## Supplementary Materials

The following supporting information can be downloaded at: https://www.mdpi.com/article/10.3390/ijms27156731/s1.

## Abbreviations

The following abbreviations are used in this manuscript:

AI	Artificial insemination
AIC	Akaike information criterion
ANOVA	Analysis of variance
CASA	Computer-aided sperm analysis
COC	Cumulus–oocyte complex
CR	Cleavage rate
DMSO	Dimethyl sulfoxide
EDR	Embryo development rate
EGFR	Epidermal growth factor receptor
FZ3	Fluo-Zin 3 AM (zinc-specific fluorescent probe)
IBFC	Image-based flow cytometry
IETS	International Embryo Technology Society
IVC	In vitro culture
IVF	In vitro fertilization
IVP	In vitro production
MAT	Maturation
MII	Metaphase II
OLS	Ordinary least squares
PI	Propidium iodide (plasma membrane integrity marker)
PNA	Peanut agglutinin (Arachis hypogaea lectin; acrosome status marker)
R^2^	Coefficient of determination
RMS	Root mean square
SEM	Standard error of the mean
SOF	Synthetic oviduct fluid
TALP	Tyrode’s albumin lactate pyruvate medium
TCM-199	Tissue culture medium 199
Zn^2+^	Zinc ion
ZnR/GPR39	Zinc receptor/G protein-coupled receptor 39
