# Supplementary material for: Bovine In Vitro Fertilization Embryo Development Predicted by Capacitation-Induced Zinc Ion Flux and Acrosome Remodeling
Source: Int J Mol Sci. 2026 Jul 28;27(15):6731. doi: 10.3390/ijms27156731 (PMC13466380; doi:10.3390/ijms27156731)
Supplement: Supplementary file 1 [file ijms-27-06731-s001.zip › ijms-4467192-supplementary.pdf]

International Journal of *Molecular Sciences*

MDPI

Supplementary Materials

Bovine in vitro fertilization embryo development predicted by capacitation-induced zinc ion flux and acrosome remodeling

Kourtney Jimmerson, Marianna Jahnke, Emma Keller, Austin Putz, Daniela Demetrio, Tyler Dohlman, Karl Kerns

Table S1. Descriptive statistics of cleavage and embryo development rates by bull. Mean, SD, SEM, minimum, and maximum for cleavage rate (CR; IVC to cleavage) and embryo development rate (EDR; maturation to Day 7 blastocyst) for each of 15 bulls across five IVF replicates (n = 5 per bull).

Bull	n	CR Mean (%)	CR SD	CR SEM	CR Min (%)	CR Max (%)	EDR Mean (%)	EDR SD	EDR SEM	EDR Min (%)	EDR Max (%)
1	5	51.7	16.8	7.5	26.7	65.5	22.7	14.2	6.4	10.0	46.7
2	5	58.0	17.7	7.9	30.0	77.8	26.0	21.4	9.6	6.7	60.0
3	5	69.5	12.8	5.7	55.6	86.4	30.7	13.6	6.1	20.0	50.0
4	5	59.0	13.7	6.1	36.7	71.4	18.7	8.4	3.7	10.0	26.7
5	5	72.6	18.5	8.3	53.3	92.9	32.0	14.6	6.5	13.3	46.7
6	5	68.0	11.5	5.1	55.2	81.5	26.0	17.5	7.8	0.0	46.7
7	5	39.4	16.5	7.4	25.0	66.7	12.0	16.4	7.3	0.0	40.0
8	5	74.0	3.0	1.3	70.4	78.6	22.0	13.2	5.9	0.0	33.3
9	5	76.3	6.1	2.7	66.7	81.5	40.0	23.5	10.5	20.0	70.0
10	5	35.3	10.4	4.7	23.3	51.9	21.3	15.4	6.9	6.7	46.7
11	5	45.9	26.4	11.8	7.7	73.7	15.3	14.6	6.5	0.0	36.7
12	5	43.2	19.5	8.7	20.7	69.2	21.3	9.6	4.3	6.7	33.3
13	5	61.4	15.9	7.1	50.0	82.8	29.3	12.8	5.7	13.3	43.3
14	5	31.5	10.3	4.6	20.0	46.7	7.3	3.7	1.6	3.3	10.0
15	5	59.8	13.0	5.8	37.0	69.0	25.3	6.9	3.1	16.7	33.3

Table S2. Coefficient of determination (R^2) for predictive models of IVF outcomes, corresponding to Figure 4. Rows are the five embryo development endpoints; columns are predictive models grouped by predictor domain and timepoint. Abbreviations: R^2, coefficient of determination; CASA, computer-aided sperm analysis (motility parameters); Bio, biomarkers (zinc signatures, acrosome remodeling [PNA], and plasma membrane integrity [PI]); Both, CASA and biomarker predictors combined; IVC, in vitro culture; 0 h, baseline (thaw); 3 h, after 3 h of in vitro capacitation; 0 & 3 h, both timepoints combined; Mixed Marginal and Mixed Conditional, the marginal (fixed effects only) and conditional (fixed plus random effects) R^2 from linear mixed models with bull and replicate as random effects. All ordinary least squares (OLS) models used stepwise Akaike information criterion (AIC) selection. The newly added single-timepoint, single-domain columns (CASA 0 h, CASA 3 h, Bio 0 h, Bio 3 h) are shown as tracked insertions.

Response	CASA 0 h	CASA 3 h	CASA 0 & 3 h	Bio 0 h	Bio 3 h	Bio 0 & 3 h	Both 0 h	Both 3 h	Both 0 & 3 h	Mixed Marginal	Mixed Conditional
Maturation to Day 7	0.103	0.058	0.188	0.131	0.078	0.253	0.153	0.231	0.482	0.384	0.487
IVC to Day 7	0.129	0.135	0.156	0.156	0.127	0.290	0.181	0.279	0.500	0.414	0.461
Cleavage to Day 7	0.000	0.090	0.098	0.027	0.075	0.212	0.081	0.156	0.333	0.298	0.413
Maturation to Cleavage	0.207	0.037	0.207	0.258	0.132	0.312	0.332	0.350	0.372	0.287	0.428
IVC to Cleavage	0.300	0.105	0.358	0.364	0.210	0.430	0.470	0.480	0.565	0.509	0.552

Table S3. Variable importance scores for predictive models of IVF outcomes (corresponds to Figure 6). Scores are scaled absolute t-statistics from stepwise-selected OLS models (both domains, 0 + 3 h). 0 Hr = measured at thaw; Delta = change from 0 to 3 h of capacitation incubation.

Trait	Variable Importance Score	Response
Signature 2 (0 hr)	4.301	Maturation to Day 7
PI+ (0 hr)	3.699	Maturation to Day 7
Signature 1 (Delta)	2.641	Maturation to Day 7
Slow Motility (Delta)	2.438	Maturation to Day 7
Progressive Motility (Delta)	2.427	Maturation to Day 7
Progressive Circular Motility (Delta)	2.425	Maturation to Day 7
PI+ (Delta)	2.42	Maturation to Day 7
Rapid Motility (Delta)	2.415	Maturation to Day 7
Coefficient Variation (0 hr)	2.376	Maturation to Day 7
PNA+++ (0 hr)	2.254	Maturation to Day 7
PNA+ (Delta)	2.211	Maturation to Day 7
Signature 4 (Delta)	2.136	Maturation to Day 7
Signature 5 (0 hr)	2.103	Maturation to Day 7
PI- (Delta)	1.763	Maturation to Day 7
Slow Motility (0 hr)	1.378	Maturation to Day 7
Total Motility (0 hr)	1.37	Maturation to Day 7
Local Motility (0 hr)	1.361	Maturation to Day 7
Rapid Motility (0 hr)	1.359	Maturation to Day 7
Progressive Circular Motility (0 hr)	1.332	Maturation to Day 7
Signature 2 (0 hr)	3.558	IVC to Day 7
Signature 1 (Delta)	2.751	IVC to Day 7
Progressive Motility (0 hr)	2.448	IVC to Day 7
Progressive Motility (Delta)	2.277	IVC to Day 7
Signature 4 (Delta)	2.228	IVC to Day 7
Slow Motility (0 hr)	2.023	IVC to Day 7
PI- (0 hr)	1.948	IVC to Day 7
Coefficient Variation (Delta)	1.851	IVC to Day 7
PNA++++ (0 hr)	1.822	IVC to Day 7
Total Motility (Delta)	1.63	IVC to Day 7
Local Motility (Delta)	1.623	IVC to Day 7
PNA++ (0 hr)	1.579	IVC to Day 7
Slow Motility (Delta)	1.521	IVC to Day 7
Progressive Circular Motility (Delta)	1.51	IVC to Day 7
Rapid Motility (Delta)	1.501	IVC to Day 7
PNA+++ (0 hr)	1.464	IVC to Day 7
PNA+ (Delta)	1.355	IVC to Day 7
Signature 5 (0 hr)	1.296	IVC to Day 7
Sig 1 (Delta)	3.283	Cleavage to Day 7
Progressive Motility (Delta)	2.795	Cleavage to Day 7
Signature 2 (0 hr)	2.699	Cleavage to Day 7
PNA++++ (0 hr)	2.572	Cleavage to Day 7
Signature 4 (Delta)	2.399	Cleavage to Day 7
PNA+ (Delta)	2.354	Cleavage to Day 7
Total Motility (Delta)	2.24	Cleavage to Day 7
Local Motility (Delta)	2.236	Cleavage to Day 7
PNA+++ (0 hr)	1.824	Cleavage to Day 7
Signature 4 (0 hr)	1.669	Cleavage to Day 7
Slow Motility (Delta)	1.496	Cleavage to Day 7
Progressive Circular Motility (Delta)	1.492	Cleavage to Day 7
Rapid Motility (Delta)	1.479	Cleavage to Day 7

Progressive Motility (0 hr)	1.298	Cleavage to Day 7
Local Motility (0 hr)	1.296	Cleavage to Day 7
Total Motility (0 hr)	1.295	Cleavage to Day 7
Slow Motility (0 hr)	1.293	Cleavage to Day 7
PNA++ (0 hr)	2.833	Maturation to Cleavage
Local Motility (Delta)	2.678	Maturation to Cleavage
Signature 4 (0 hr)	2.594	Maturation to Cleavage
Signature 3 (0 hr)	2.455	Maturation to Cleavage
Slow Motility (Delta)	2.438	Maturation to Cleavage
Rapid Motility (Delta)	2.423	Maturation to Cleavage
Total Motility (Delta)	2.361	Maturation to Cleavage
Signature 2 (0 hr)	1.483	Maturation to Cleavage
Signature 5 (0 hr)	1.326	Maturation to Cleavage
Signature 3 (0 hr)	3.862	IVC to Cleavage
PNA++ (0 hr)	3.473	IVC to Cleavage
Signature 4 (0 hr)	3.248	IVC to Cleavage
Local Motility (Delta)	2.874	IVC to Cleavage
Signature 2 (0 hr)	2.436	IVC to Cleavage
Total Motility (Delta)	1.985	IVC to Cleavage
PNA+ (0 hr)	1.936	IVC to Cleavage
Coefficient Variation (Delta)	1.845	IVC to Cleavage
Slow Motility (Delta)	1.802	IVC to Cleavage
Rapid Motility (Delta)	1.624	IVC to Cleavage
Local Motility (0 hr)	1.328	IVC to Cleavage
Total Motility (0 hr)	1.317	IVC to Cleavage
Progressive Motility (0 hr)	1.314	IVC to Cleavage

Table S4. Partial (nested) F-tests comparing each stepwise-selected combined (0 + 3 h) predictive model with a reduced model retaining only its baseline (0 h) terms, for each embryo development endpoint. A significant result indicates that the capacitation-induced change (Δ, 0→3 h) predictors jointly improved model fit. F, F-statistic; df1, numerator degrees of freedom (number of Δ predictors); df2, residual degrees of freedom; P, significance.

**Endpoint**	**F**	**df1**	**df2**	**P**
Maturation to Day 7	3.52	9	55	0.002
IVC to Day 7	3.35	10	56	0.002
Cleavage to Day 7	3.71	9	57	0.001
Maturation to Cleavage	2.65	4	65	0.041
IVC to Cleavage	3.36	5	61	0.010
